# Supplementary figures and images for: Precision pharmacological reversal of strain-specific diet-induced metabolic syndrome in mice informed by epigenetic and transcriptional regulation
Source: PLoS Genet. 2023 Oct 23;19(10):e1010997. doi: 10.1371/journal.pgen.1010997 (PMC10621921; doi:10.1371/journal.pgen.1010997)

■ positive z-score    □ z-score = 0    ■ negative z-score    ■ no activity pattern available

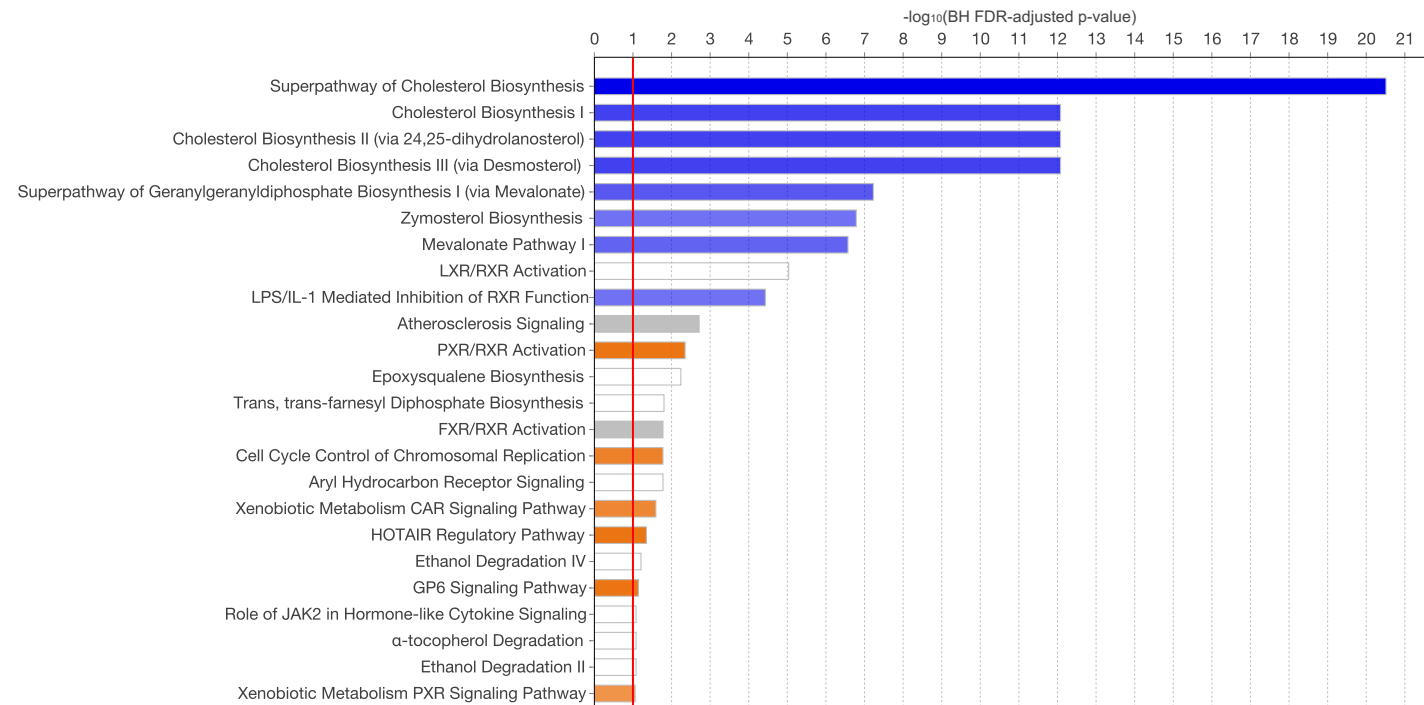

Supplement: S1 Fig — IPA results showing enriched pathways among differentially expressed genes between BL6 mice on the American vs. standard diet. All significantly enriched pathways with BH FDR-adjusted p-values < 0.1 are shown. (PDF) [file pgen.1010997.s001.pdf]

■ positive z-score  
 ■ z-score = 0  
 ■ negative z-score  
 ■ no activity pattern available

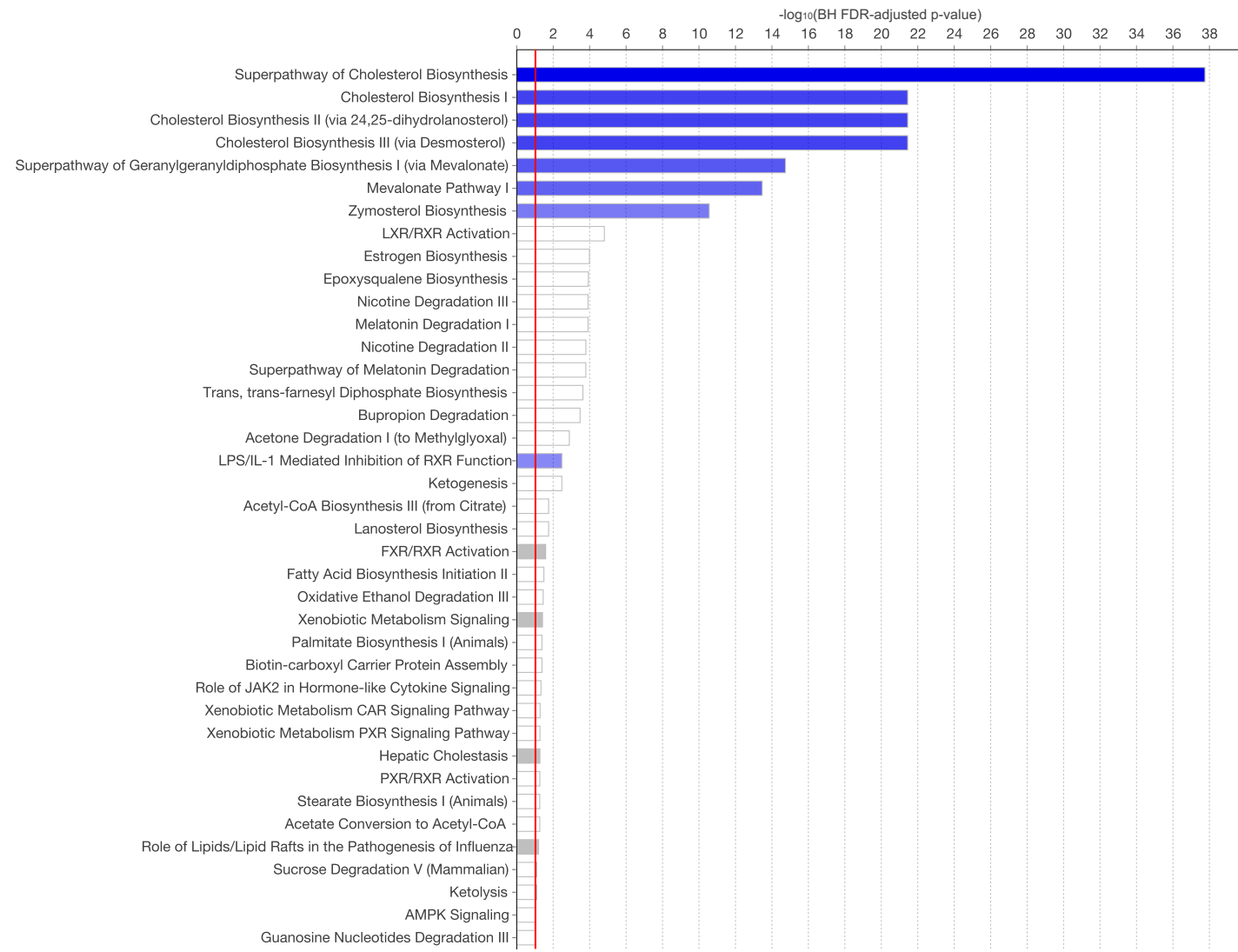

Supplement: S2 Fig — IPA results showing enriched pathways among differentially expressed genes between A/J mice on the American vs. standard diet. All significantly enriched pathways with BH FDR-adjusted p-values < 0.1 are shown. (PDF) [file pgen.1010997.s002.pdf]

■ positive z-score
 ■ z-score = 0
 ■ negative z-score
 ■ no activity pattern available

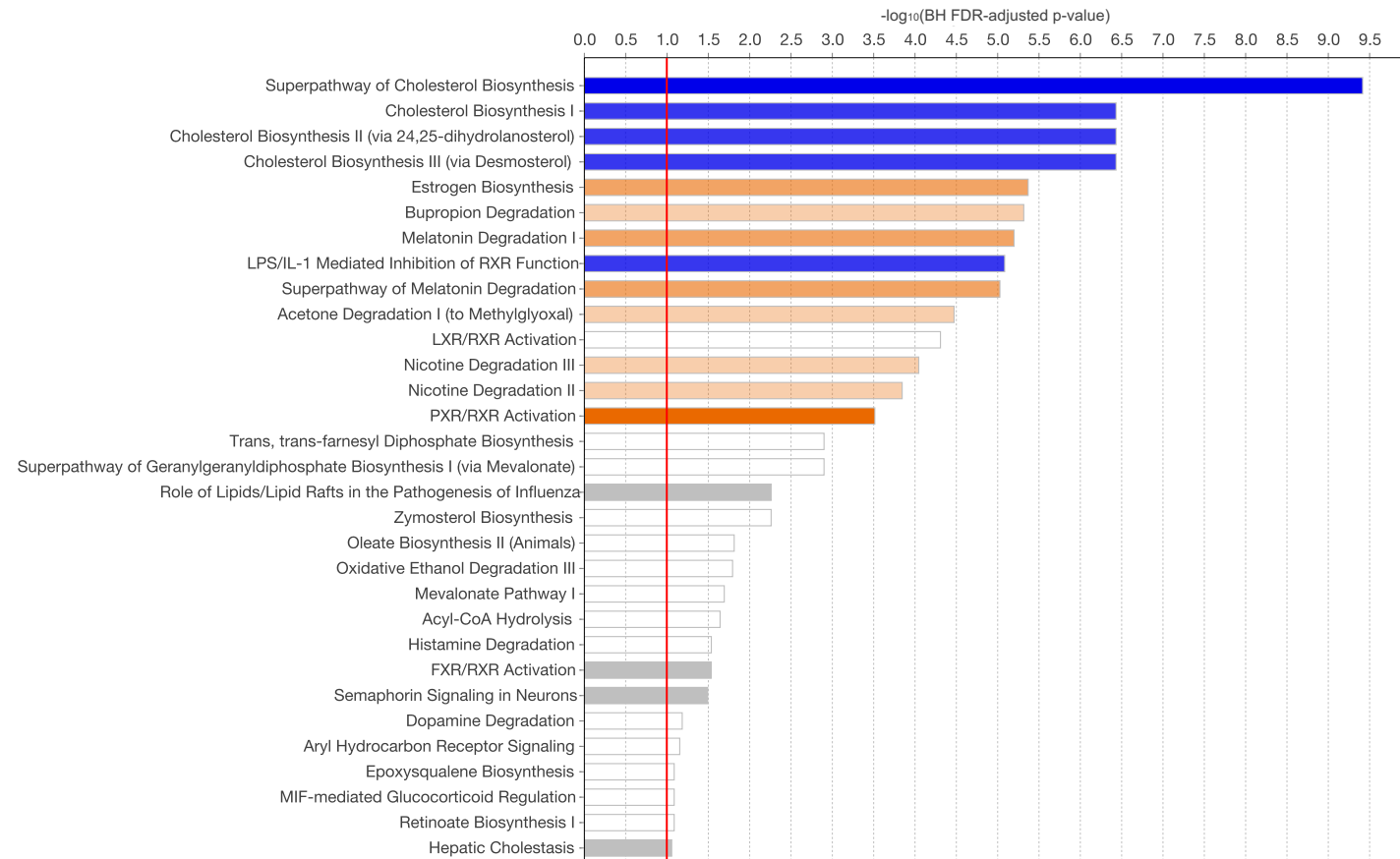

Supplement: S3 Fig — IPA results showing enriched pathways among differentially expressed genes between NOD mice on the American vs. standard diet. All significantly enriched pathways with BH FDR-adjusted p-values < 0.1 are shown. (PDF) [file pgen.1010997.s003.pdf]

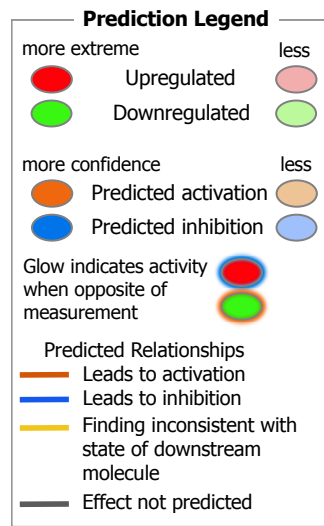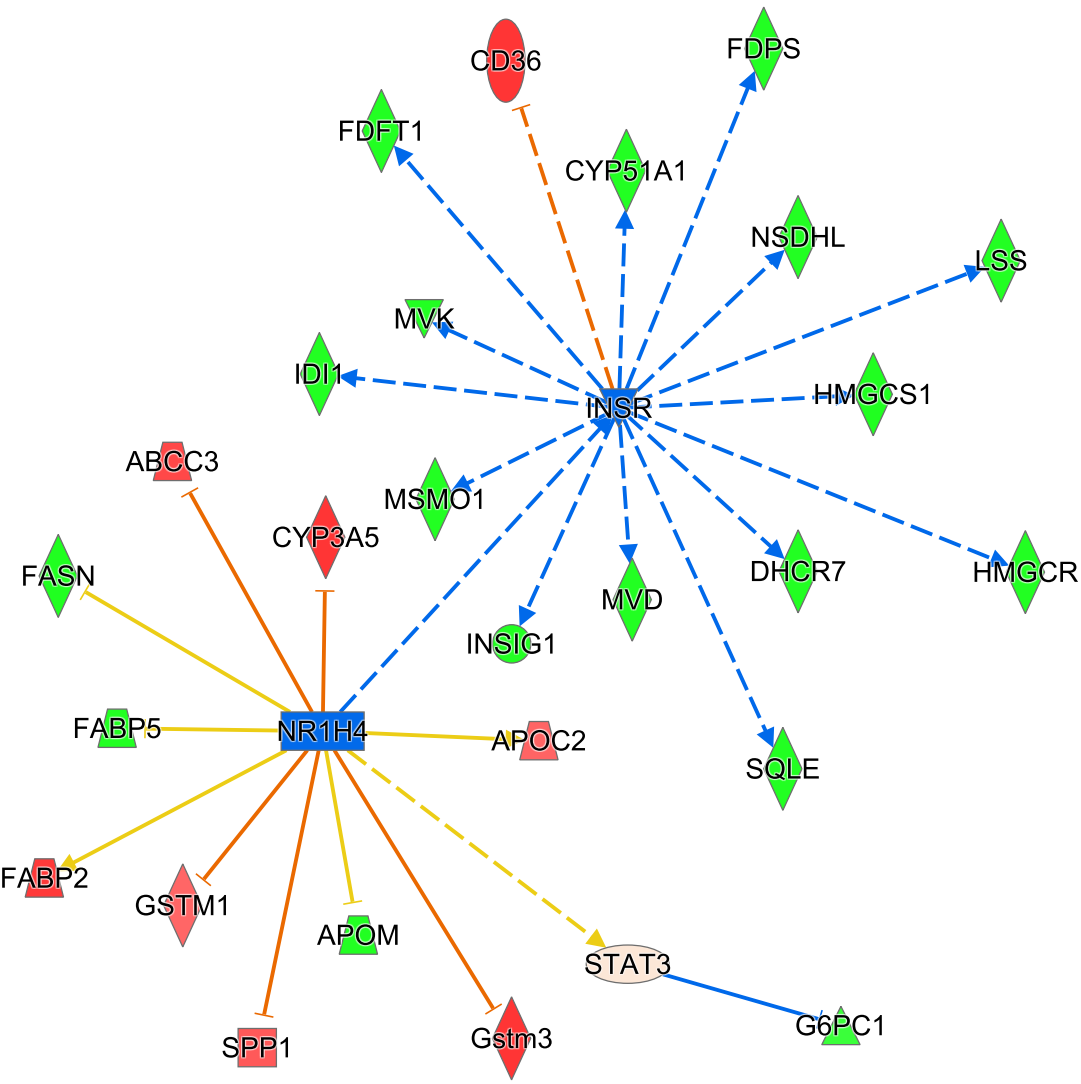

Supplement: S4 Fig — Network graph depicting the overlay of differentially expressed genes between BL6 mice on the American vs. standard diet and associated IPA network activity predictions on the FXR (Nr1h4) master regulatory network. (PDF) [file pgen.1010997.s004.pdf]

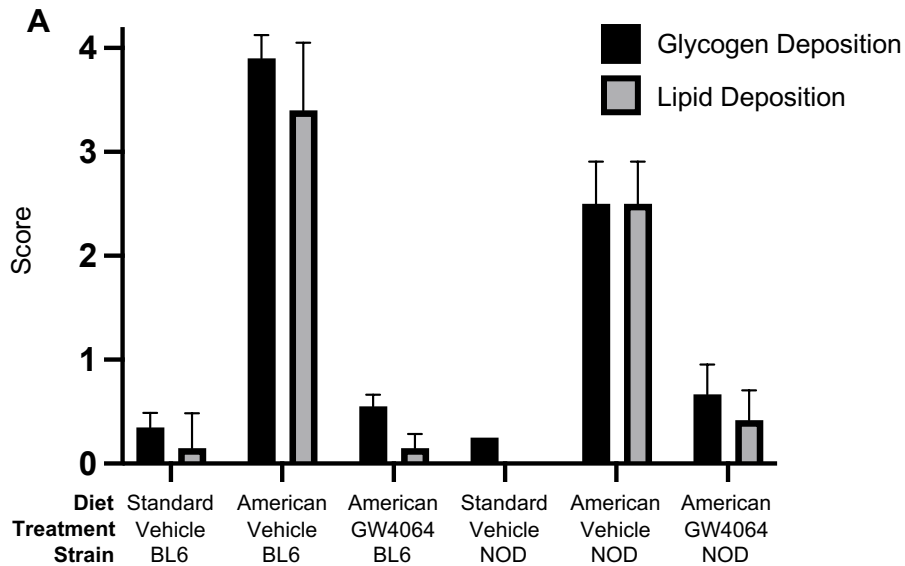

Standard + Vehicle

American + Vehicle

American + GW4064

BL6

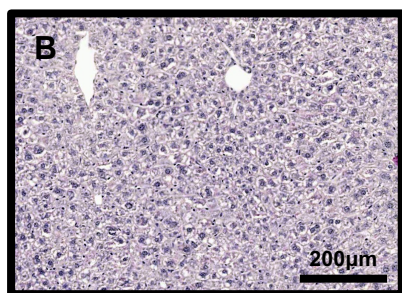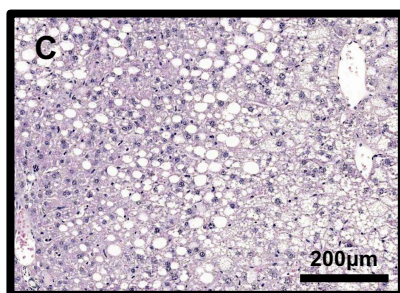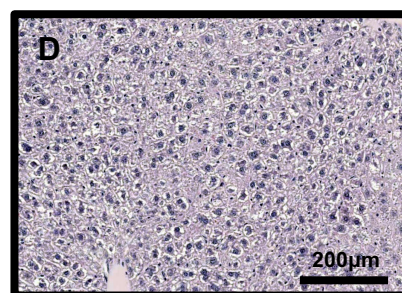

NOD

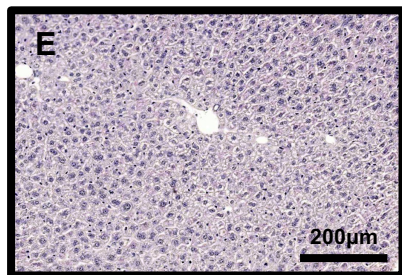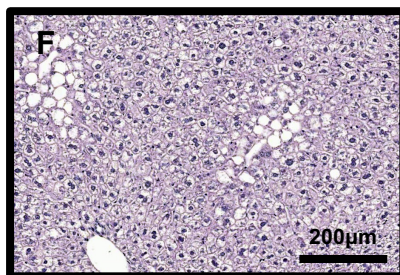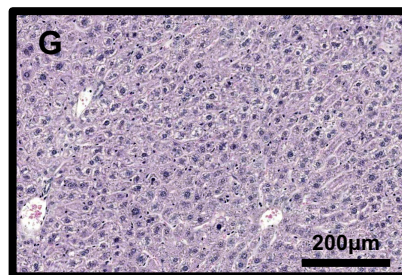

PAS

PAS-D

BL6

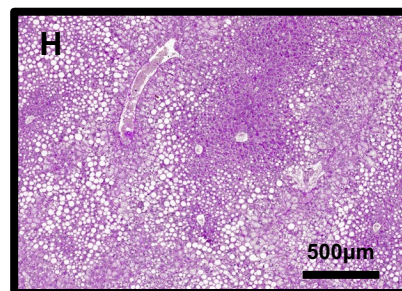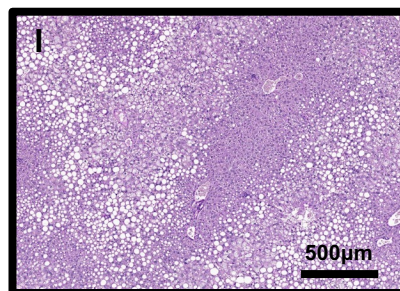

NOD

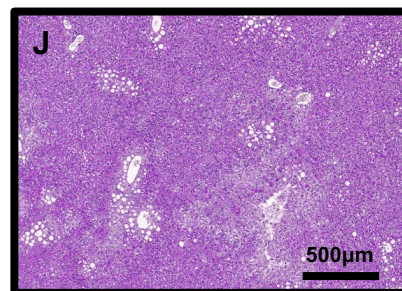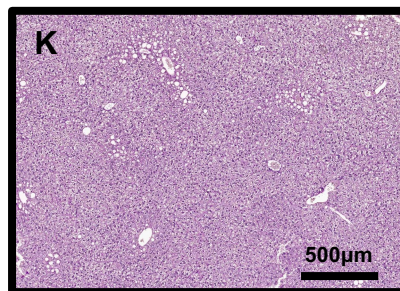

Supplement: S5 Fig — (A) Scores for glycogen and lipid deposition in each diet/treatment group. (B-G) Representative liver histology images for: (B) BL6 on the standard diet with vehicle; (C) BL6 on the American diet with vehicle; (D) BL6 on the American diet with GW4064 treatment; (E) NOD on the standard diet with vehicle; (F) NOD on the American diet with vehicle; and (G) NOD on the American diet with GW4064 treatment. Bars in (B-G) are 200μm. (H and J) Sections of liver stained with PAS, rendering glycogen as magenta for: (H) BL6 on the American diet with vehicle; (J) NOD on the American diet with vehicle. (I and K) Sections of liver that were digested with diastase and stained with PAS-D reagent, confirming loss of magenta-colored glycogen for: (I) BL6 on the American diet with vehicle; (K) NOD on the American diet with vehicle. Bars in (H-K) are 500μm. (PDF) [file pgen.1010997.s005.pdf]

■ positive z-score   ■ z-score = 0   ■ negative z-score   ■ no activity pattern available

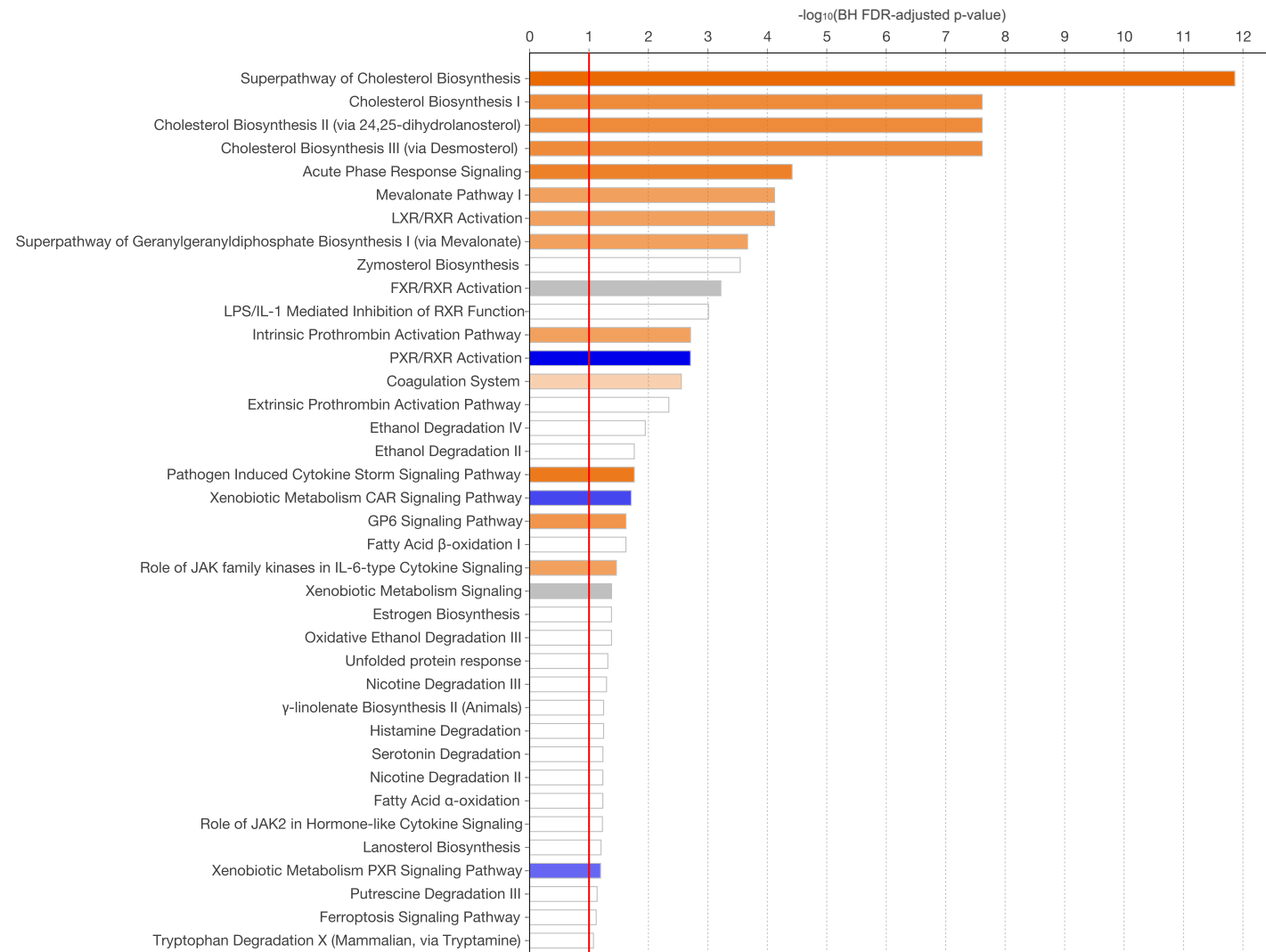

Supplement: S6 Fig — IPA results showing enriched pathways among differentially expressed genes between BL6 mice given the American diet + GW4064 vs. American diet + vehicle. All significantly enriched pathways with BH FDR-adjusted p-values < 0.1 are shown. (PDF) [file pgen.1010997.s006.pdf]

■ positive z-score   ■ z-score = 0   ■ negative z-score   ■ no activity pattern available

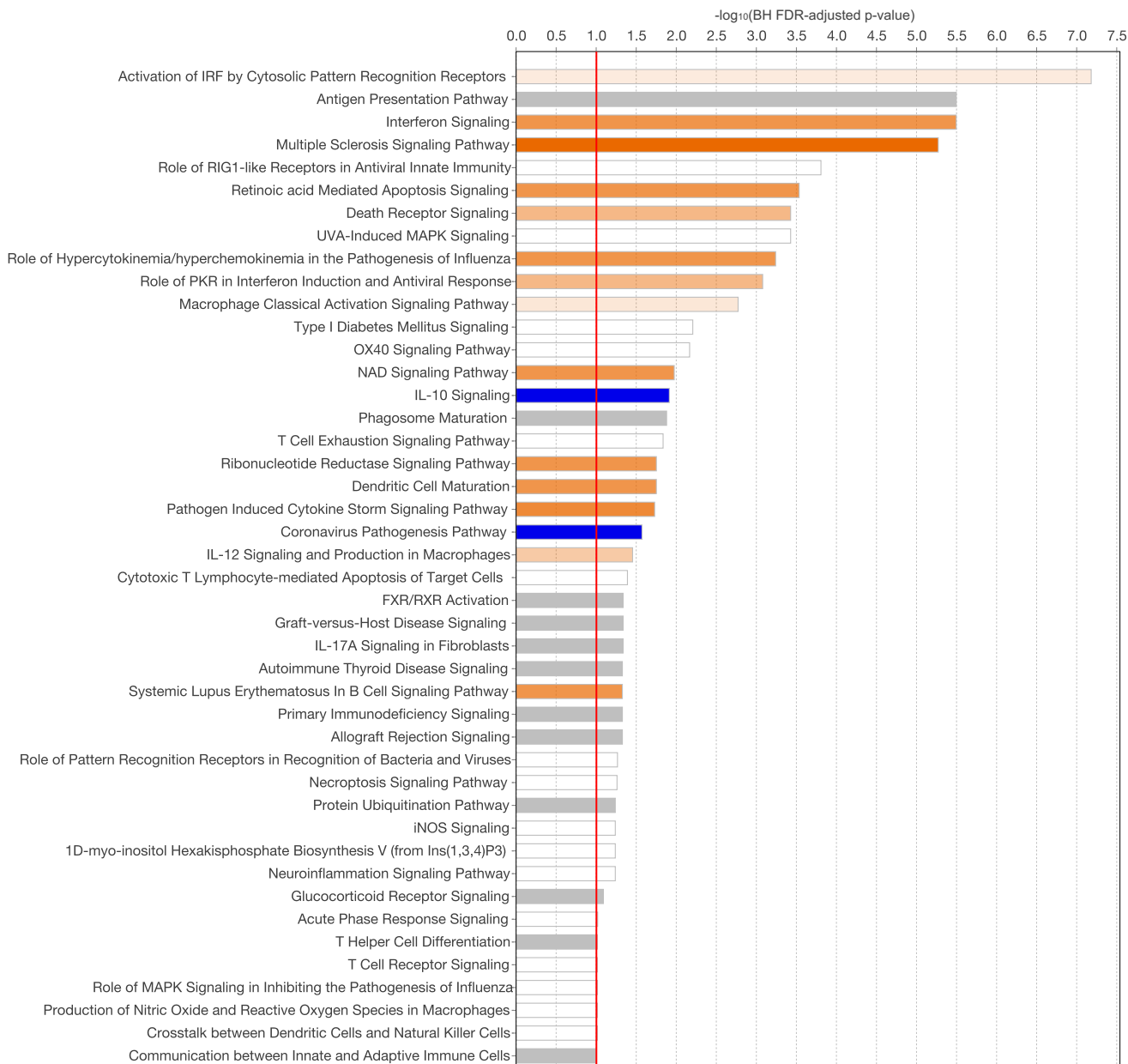

Supplement: S7 Fig — IPA results showing enriched pathways among differentially expressed genes between NOD mice given the American diet + GW4064 vs. American diet + vehicle. All significantly enriched pathways with BH FDR-adjusted p-values < 0.1 are shown. (PDF) [file pgen.1010997.s007.pdf]

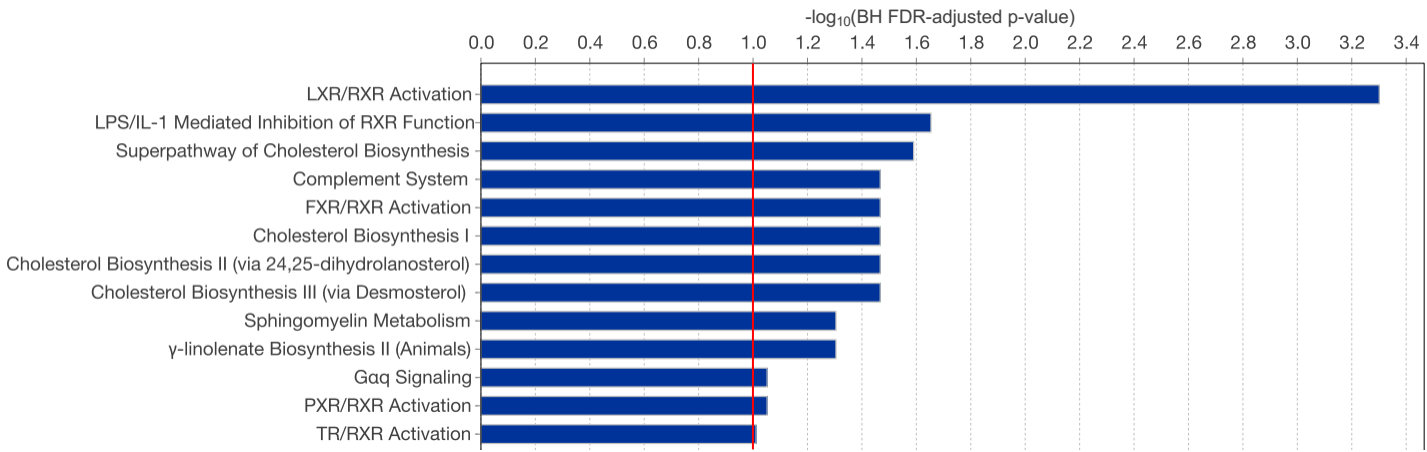

Supplement: S8 Fig — IPA results showing enriched pathways among differentially methylated region-associated genes between BL6 mice on the American vs. standard diet. All significantly enriched pathways with BH FDR-adjusted p-values < 0.1 are shown. (PDF) [file pgen.1010997.s008.pdf]

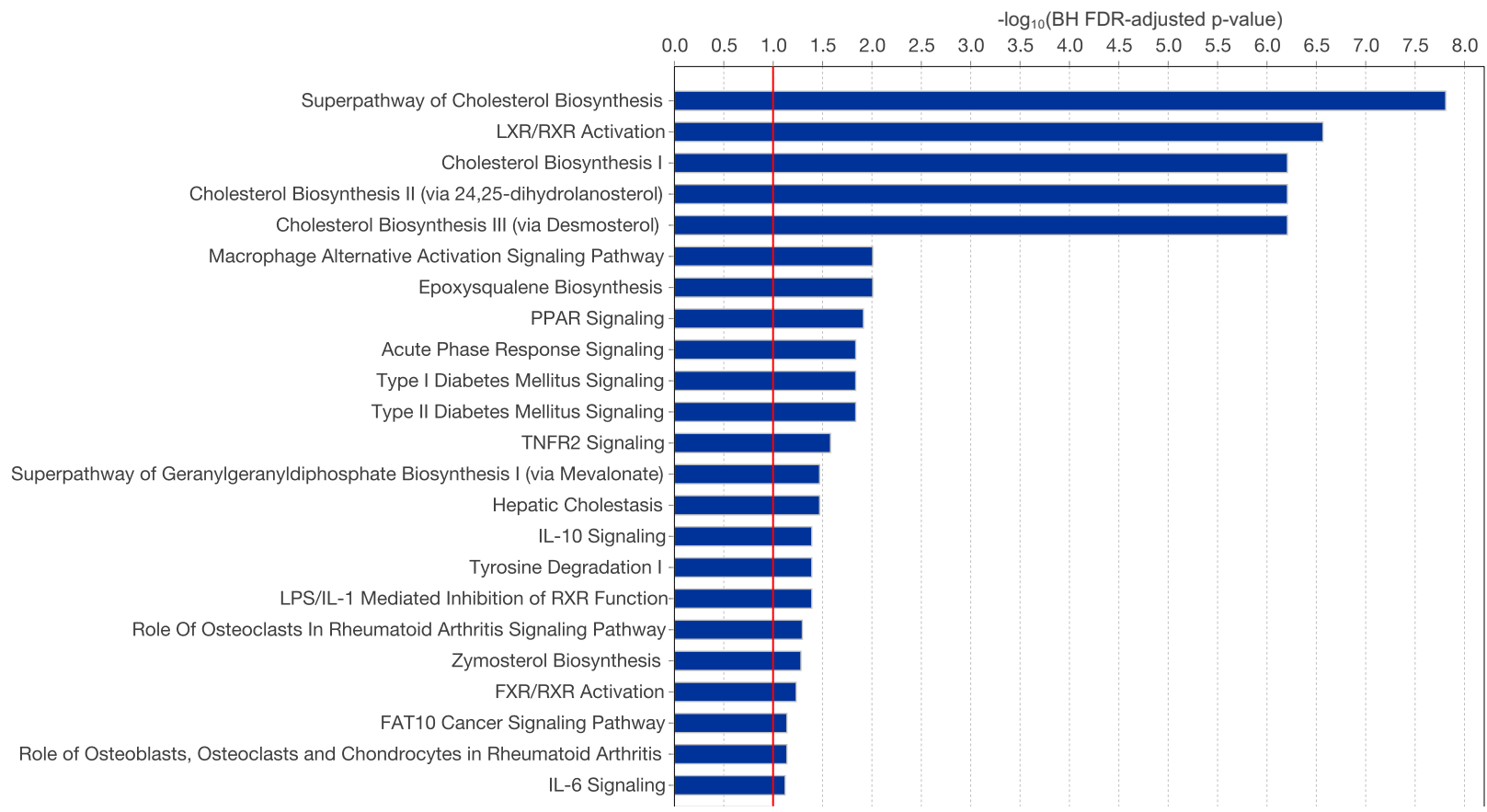

Supplement: S9 Fig — IPA results showing enriched pathways among differentially methylated region-associated genes between A/J mice on the American vs. standard diet. All significantly enriched pathways with BH FDR-adjusted p-values < 0.1 are shown. (PDF) [file pgen.1010997.s009.pdf]

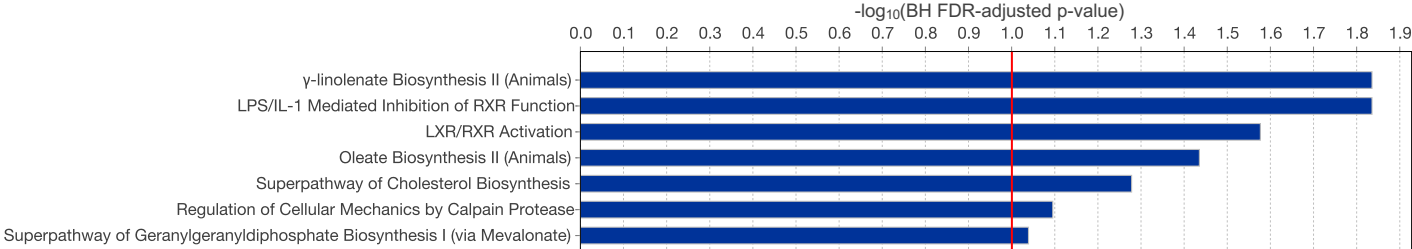

Supplement: S10 Fig — IPA results showing enriched pathways among differentially methylated region-associated genes between NOD mice on the American vs. standard diet. All significantly enriched pathways with BH FDR-adjusted p-values < 0.1 are shown. (PDF) [file pgen.1010997.s010.pdf]
